# Potential Molecular Mechanisms of Chaihu-Shugan-San in Treatment of Breast Cancer Based on Network Pharmacology

**DOI:** 10.1155/2020/3670309

**Published:** 2020-09-25

**Authors:** Kunmin Xiao, Kexin Li, Sidan Long, Chenfan Kong, Shijie Zhu

**Affiliations:** ^1^Graduate School, Beijing University of Chinese Medicine, Beijing 100029, China; ^2^Department of Oncology, Wangjing Hospital, China Academy of Chinese Medical Sciences, Beijing 100102, China

## Abstract

Breast cancer is one of the most common cancers endangering women's health all over the world. Traditional Chinese medicine is increasingly recognized as a possible complementary and alternative therapy for breast cancer. Chaihu-Shugan-San is a traditional Chinese medicine prescription, which is extensively used in clinical practice. Its therapeutic effect on breast cancer has attracted extensive attention, but its mechanism of action is still unclear. In this study, we explored the molecular mechanism of Chaihu-Shugan-San in the treatment of breast cancer by network pharmacology. The results showed that 157 active ingredients and 8074 potential drug targets were obtained in the TCMSP database according to the screening conditions. 2384 disease targets were collected in the TTD, OMIM, DrugBank, GeneCards disease database. We applied the Bisogenet plug-in in Cytoscape 3.7.1 to obtain 451 core targets. The biological process of gene ontology (GO) involves the mRNA catabolic process, RNA catabolic process, telomere organization, nucleobase-containing compound catabolic process, heterocycle catabolic process, and so on. In cellular component, cytosolic part, focal adhesion, cell-substrate adherens junction, and cell-substrate junction are highly correlated with breast cancer. In the molecular function category, most proteins were addressed to ubiquitin-like protein ligase binding, protein domain specific binding, and Nop56p-associated pre-rRNA complex. Besides, the results of the KEGG pathway analysis showed that the pathways mainly involved in apoptosis, cell cycle, transcriptional dysregulation, endocrine resistance, and viral infection. In conclusion, the treatment of breast cancer by Chaihu-Shugan-San is the result of multicomponent, multitarget, and multipathway interaction. This study provides a certain theoretical basis for the treatment of breast cancer by Chaihu-Shugan-San and has certain reference value for the development and application of new drugs.

## 1. Introduction

Breast cancer is one of the most common cancers endangering women's health all over the world. The GLOBO-CAN 2018 statistics show alarming results that there are 8.6 million new cases of female cancer and 4.2 million female cancer deaths worldwide. The proportion of breast cancer is 24.2% and 15.0%, respectively, ranking first in female cancer incidence and death [1]. It is predicted that, by the 2050s, the global incidence of breast cancer will reach nearly 3,200,000 new cases of breast cancer each year. These datasets reflect the high incidence of breast cancer and the urgent global need for breast cancer prevention and treatment measures [2].

Traditional Chinese medicine (TCM) has a long history in the etiology, pathogenesis, prevention, and treatment of breast cancer. According to the principle of TCM syndrome differentiation and treatment, the clinical syndrome of breast cancer is mainly “Liver-Qi” stagnation. Chaihu-Shugan-San is one of the classical prescriptions for the treatment of “Liver-Qi” stagnation. It has the effect of soothing “Liver-Qi.” It has a history of 485 years and is widely used in clinical practice [3–5]. Chaihu-Shugan-San includes seven kinds of traditional Chinese medicine such as Bupleurum chinense DC (Chinese name: Chaihu), Radix Paeoniae Alba (Chinese name: Baishao), Citrus reticulata Blanco (Chinese name Chenpi), Cyperus rotundus L (Chinese name: Xiangfu), Glycyrrhiza uralensis Fisch (Chinese name: Gancao), Citrus aurantium L (Chinese name: Zhiqiao), and Ligusticum chuanxiong Hort (Chinese name: Chuanxiong) [6]. Traditional Chinese medicine meridian tropism is one of the core components of the theory of TCM. According to the theory of TCM, the characteristics of the selective distribution of the effective components of traditional Chinese medicine in the body are basically consistent with the relationship between the viscera and viscera of the corresponding meridian tropism. Chaihu, Chenpi, Xiangfu, Chuanxiong, and Baishao in Chaihu-Shugan-San belong to the liver meridian, which can soothe the liver and regulate Qi well. In traditional Chinese medicine theory, the main location of breast cancer is in the liver and often related to the spleen and kidney in the process of disease. Chaihu-Shugan-San meridian attribution is consistent with breast cancer meridian attribution and can better play the therapeutic effect.

As a classical prescription, Chaihu-Shugan-San has been widely studied in pharmacology. Chaihu-Shugan-San has the pharmacological effects of antidepressant [7], regulation of neuro-endocrine-immune network [8, 9], anti-inflammation, antioxidative stress [10], lipid-lowering and glucose-lowering, and antifibrosis [11]. In terms of antitumor, in a study of 86 patients with stage III breast cancer, the control group was given a standardized CAF regimen (cyclophosphamide + doxorubicin + 5-fluorouracil), and the experimental group was treated with Chaihu-Shugan-San on the basis of CAF. The short-term effective rate of the observation group was significantly higher than that of the control group (81.4% vs. 58.14%); the Karnofsky improvement rate of the observation group was significantly higher than that of the control group (48.84% vs. 34.88%) [12]. As a safe complementary alternative therapy, Chaihu-Shugan-San combined with other chemotherapy regimens can improve the therapeutic effect, alleviate the myelosuppression caused by chemotherapeutic drugs, and improve the prognosis of breast cancer patients [13, 14]. However, the mechanism of Chaihu-Shugan-San in the treatment of breast cancer remains to be further explored.

Classical prescriptions are currently the preferred way to treat diseases in TCM clinic, but they lack a scientific basis to reasonably explain the mechanism of TCM prescriptions from the whole to the local level or from the cellular to the molecular level [15]. Network pharmacology is an emerging discipline based on the integration of systems biology, molecular biology, pharmacology, and a variety of network computing platforms in the context of the era of big data, which can more directly explain the association between TCM prescriptions and diseases [16]. Therefore, this study constructed a multidimensional network of “ingredient-target-pathway” through network pharmacology to explore the potential molecular mechanism of Chaihu-Shugan-San in the treatment of breast cancer and to provide a certain theoretical basis for Chaihu-Shugan-San in the treatment of breast cancer.

## 2. Materials and Methods

### 2.1. Screening of Active Components and Target Prediction in Chaihu-Shugan-San

In this study, the chemical components of the seven herbs were searched on Traditional Chinese Medicine Systems Pharmacology Database and Analysis Platform (TCMSP, http://tcmspw.com/tcmsp.php, updated on May 31, 2014) [17]. Search keywords are Chaihu, Baishao, Chenpi, Xiangfu, Gancao, Zhiqiao, and Chuanxiong, and only oral bioavailability (OB) ≥30% and drug-likeness (DL) ≥0.18 were considered in this study. The Canonical SMILES sequence of the compound was searched in the PubChem database (https://pubchem.ncbi.nlm.nih.gov/) [18], and this sequence was used to predict the target of the compound in the Swiss Target Prediction online database (http://www.swisstargetprediction.ch/) [19] and collect target protein gene names and UniProt ID in the prediction results.

### 2.2. Network Construction of Components and Targets

The chemical composition and potential targets of the above Chaihu-Shugan-San were uploaded to Cytoscape 3.7.1 [20] software to build up the component-target network. In the network, the degree centrality (DC) represents the number of nodes in the network that directly interacts with the node. The greater the degree, the more the biological functions it participates in; the betweenness centrality (BC) refers to the proportion of the number of nodes passing through the shortest path in the network, and the larger the BC is, the more influential the node is. Closeness centrality (CC) reflects the degree of proximity between nodes, and the reciprocal of the shortest path distance from one node to other nodes is CC. The closer the nodes are, the larger the CC is; the average shortest path length (ASPL) is the average of the shortest path length between all points in the network. The smaller the average path of a node, the more crucial this node is in the network.

### 2.3. Prediction of Breast Cancer Targets

With “breast cancer” or “malignant breast tumors” as keywords, we searched in Online Mendelian Inheritance in Man (OMIM, http://www.omim.org/, updated on May 4, 2018) [21], DrugBank (https://www.drugbank.ca/, version 5.1.6, updated on Apr 22, 2020) [22], Therapeutic Target Database (TTD, http://db.idrblab.net/ttd/, updated on Nov 11, 2019) [23], and GeneCards (https://www.genecards.org/, version 4.14.0) [24] to collect breast cancer-related targets. In the GeneCards database, the higher the score value is, the closer the relationship between the target and disease is, and the score value greater than the median is used as the screening condition to extract the key target. The above retrieval results were combined to remove duplicates and serve as the prediction target library of breast cancer.

### 2.4. Protein-Protein Interaction (PPI) Network Construction and Selection of Core Targets

The BisoGenet plug-in in Cytoscape 3.7.1 draws the PPI network and maps the Chaihu-Shugan decoction component targets and breast cancer-related disease targets into the protein interaction relationship network, using Cytoscape 3.7.1 merge two protein interaction networks, to extract the intersection of the network. Based on the intersection network, the CytoNCA plug-in [25] is used to screen out the nodes whose degree centrality (DC) is greater than 2 times the median of all nodes. After multiple screening, the core PPI network is finally obtained.

### 2.5. GO Functional Enrichment Analysis and KEGG Pathway Enrichment Analysis

The core target of PPI network selected above was imported into Metascape (https://metascape.org/gp/index.html, updated on March 20, 2020) [26] database for KEGG (Kyoto Encyclopedia of Genes and Genomes) pathway analysis and GO (Gene Ontology) biological process enrichment analysis. Parameter is set to min overlap >3, *p* value cutoff <0.01, and min enrichment >1.5. Taking *p* value as parameter and sorting from small to large as screening condition, KEGG pathway and GO biological process of the top 20 eligible were selected and uploaded to OmicShare (http://www.omicshare.com/tools) platform for data visualization.

### 2.6. Constructing PPI Network of Ingredient-Disease-KEGG Pathway

The top 20 KEGG pathways, Chaihu-Shugan-San active ingredients, and disease common targets were uploaded to Cytoscape 3.7.1 software to obtain the multidimensional network diagram of component-disease -KEGG pathway.

## 3. Results

### 3.1. Active Ingredient and Target of Chaihu-Shugan-San

OB ≥30%, DL ≥0.18 as the screening conditions, after searching TCMSP database, ChaiHu-ShuGan-San obtained a total of 157 chemical components, 13 compounds from Baishao, 17 compounds from Chaihu, 5 compounds from Chenpi, 7 compounds from Chuanxiong, 5 compounds from Zhiqiao, 92 qualified compounds from Gancao, and 18 compounds from Xiangfu (as shown in Table S1 in Supplementary Materials). 8074 targets were obtained by inputting 158 chemical components into Swiss Target Prediction online database.

### 3.2. Compound-Target Network Construction

**Figure 1:**
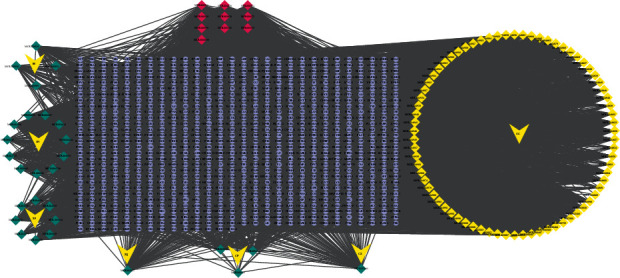
Compound-target network: green diamond nodes represent compounds, red diamond nodes represent common compounds, yellow v nodes represent herb names, and purple circular nodes represent corresponding potential targets of compounds.

The compound-target network consists of 945 nodes and 8200 edges. 29 of 157 compounds were not found in the database and not involved in the network construction (Figure 1). In this network, the average degree value is 15.647, and most of the proteins share common ligands with other proteins, which reflects the mechanism of the joint action between multicomponents and multitargets of Chaihu-Shugan-San, and conform to the characteristics of the traditional Chinese formula.  Table 1 shows the detailed topological parameters of the top 10 compounds with high DC.

### 3.3. Screening of Breast Cancer Targets

Breast cancer or malignant breast tumors were used as keywords to search in TTD, OMIM, DrugBank, and GeneCards databases. 37 disease targets were obtained from TTD database, 1163 disease targets were screened from OMIM database, 202 disease targets were screened from Drugbank database, and 1286 disease targets with score >13.96 were obtained from GeneCards database. The duplicates were deleted after merging, and 2,384 breast cancer-related targets were finally obtained.

### 3.4. Construction of PPI Network of Chaihu-Shugan-San and Disease Targets

**Figure 2:**
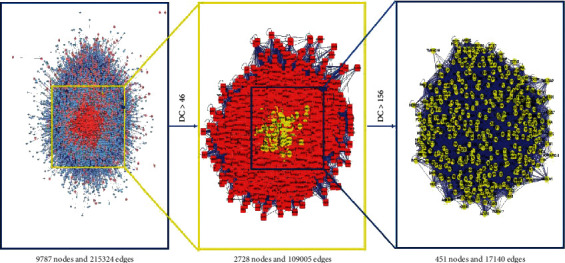
Network topology analysis of PPI.

To further explore the pharmacological mechanism of Chaihu-Shugan-San on breast cancer, Chaihu-Shugan-San and breast cancer protein were input into the BisoGenet plug-in of Cytoscape 7.2.1 software for merging. CytoNCA plug-in performs topological analysis and takes 2 times of the average degree value as the screening condition. In the first screening, a network composed of 2,728 nodes and 109,005 edges was obtained by the median DC >46. Finally, a PPI network with 451 nodes and 17,140 edges was constructed by further screening with the median DC >156. The process is shown in Figure 2. Topological parameters of the top 10 targets with high DC are shown in Table 2, and other detailed results are shown in Table S2.

### 3.5. GO Biological Process and Enrichment Analysis of KEGG Pathway

**Figure 3:**
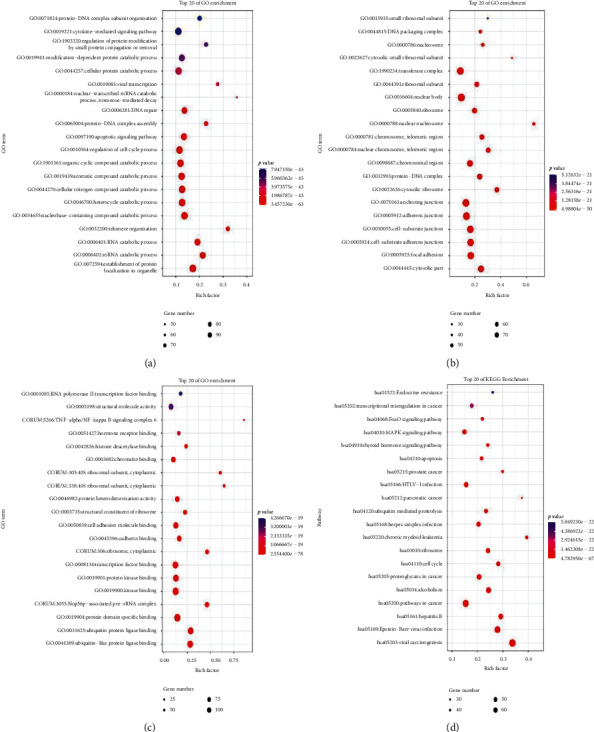
GO function enrichment analysis and enrichment analysis of KEGG signaling pathway (top 20). (a) BP. (b) CC. (c) MF. (d) KEGG.

GO biological process consists of molecular function (MF), biological process (BP), and cellular component (CC) to interpret antitumor biological processes at key targets. Kyoto Encyclopedia of Genes and Genomes (KEGG) enrichment analysis studies the key target of antitumor signaling pathways. The results of GO enrichment analysis showed that there were 2000 biological processes, 274 cell components, and 564 molecular functions. KEGG enrichment has 154 signaling pathways. According to the ranking of *p* values, the top 20 were selected to plot the bubble chart (Figure 3). The left side of each chart is the top enriched name. The color of bubbles from blue to red represents the *p* value from large to small. The larger the bubbles, the larger the gene count of the pathway. The horizontal axis represents the ratio of the pathway genes to the total input genes. The top 20 signal pathways of KEGG enrichment are shown in Table S3.

### 3.6. The Multidimensional Network of “Component-Target Disease-KEGG Signaling Pathway” Was Constructed

**Figure 4:**
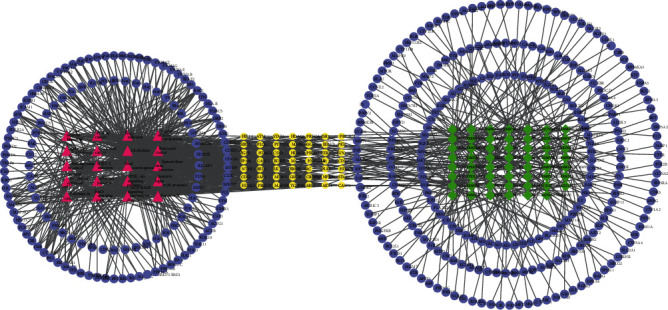
Component-disease target-KEGG signaling pathway. Green represents active ingredients of drugs; purple represents targets; yellow represents common targets; and red represents signaling pathways.

Combining the component-target network and the first 20 KEGG signaling pathway targets, a multidimensional network of “component-disease target-KEGG signaling pathway” was obtained by Cytoscape 7.2.1 software (as shown in Figure 4). The results showed that the effective components of Chaihu-Shugan-San could treat breast cancer by multitarget and multisignal pathways.

## 4. Discussion

Traditional Chinese medicine compound acts on diseases through multimolecule, multitarget, and multipathway and plays a certain therapeutic effect. Network pharmacology is a science based on the macroconnection under the background of the big data era. It systematically analyzes the molecular mechanism of action from all levels, which is consistent with the holistic view of TCM and the thought of syndrome differentiation and treatment. Chaihu-Shugan-San prescription, in which Chaihu is the monarch drug, is good at soothing the “Liver-Qi.” Xiangfu and Chuanxiong are the minister drugs, which can relieve “Liver-Qi.” Chenpi and Zhiqiao, regulating “Qi” stagnation and Baishao, nourishing “Blood” and softening the “Liver,” are adjuvants. Gancao is used as a guide medicine to reconcile various drugs. The combination of various drugs can regulate “Liver-Qi” and smooth “Qi.” Chaihu-Shugan-San has a history of more than 480 years. The basic compatibility of clinical medication is Chenpi 6 g, Chaihu 6 g, Chuanxiong 4.5 g, Xiangfu 4.5 g, Zhiqiao 4.5 g, Shaoyao 4.5 g, and Gancao 1.5 g, which is added or subtracted according to the actual situation of patients. The official preparation method is to add 250 ml of water to the herbs and boil them for 30 min. Su [27] and his team identified 33 chemical constituents in Chaihu-Shugan-San. Among them, gallic acid (source: Shaoyao), oxidized paeoniflorin (source: Shaoyao), paeoniflorin (source: Shaoyao), paeoniflorin (source: Shaoyao), glycyrrhizin (source: Gancao), naringin (source: Chenpi, Zhiqiao), hesperidin (source: Chenpi, Zhiqiao), and ferulic acid (source: Chuanxiong) had higher contents, all above 1000 mg/g [28]. Although some studies have comprehensively elucidated the treatment of depression [6, 29], nonalcoholic fatty liver disease [30, 31] and functional dyspepsia [5] by Chaihu-Shugan-San, no studies have comprehensively elucidated the mechanism of Chaihu-Shugan-San in the treatment of breast cancer. Therefore, with the aid of network pharmacology, this study analyzed the specific molecular mechanism of Chaihu-Shugan-San in the treatment of breast cancer from a microscopic perspective.

The results of network analysis showed that the active ingredients in Chaihu-Shugan-San mainly included beta-sitosterol, kaempferol, quercetin, naringenin, isorhamnetin, and nobiletin. Beta-sitosterol can promote the apoptosis of breast cancer cells by activating the Fas signaling pathway and caspase-8 activity [32] and is expected to be an orphan nutrition drug against cancer [33]. Kaempferol has shown a good affinity for PAK4 in molecular docking and is considered to be a potential inhibitor in triple-negative breast cancer [34], and kaempferol can prevent G2/M phase of the cell cycle by downregulating CDK1 in human breast cancer MDA-MB-453 cells [35], and blocking RhoA and Rac1 signaling pathways to inhibit breast cancer cell migration and invasion [36] is a powerful antioxidant inducer and can inhibit oncogene transformation and induce cancer cell apoptosis and DNA damage. Quercetin, naringenin, and isorhamnetin, such as flavonoids, can prevent breast cancer cell migration through inflammatory and apoptotic cell signaling [37, 38], and quercetin can induce autophagy by inhibiting the Akt-mTOR pathway [39].

The PPI network showed that the active components in Chaihu-Shugan-San may function through the core targets such as histone deacetylase 1 (HDAC1), huntingtin (HTT), RAC-alpha serine/threonine-protein kinase (AKT1), hepatoma-derived growth factor (HDGF), roquin-1 (RC3H1), chromobox protein homolog 8 (CBX8), histone deacetylase 2 (HDAC2), small ubiquitin-related modifier 1 (SUMO1), 40 S ribosomal protein SA (RPSA), and 60 S acidic ribosomal protein P0(RPLP0). HDAC1 plays an important role in transcriptional regulation and cell cycle progression [40]. HDAC1 can promote the proliferation and migration of breast cancer cells by activating the Snail/IL-8 signaling pathway [41]. Downregulation of HTT transcription and protein levels is a key factor in poor prognosis and metastasis development of breast cancer [42]. AKT1 is involved in the regulation of many tumor processes, including tumor proliferation, cell survival, metabolism, growth, and angiogenesis. The mutation frequency of AKT1 in Chinese breast cancer patients is 3.2%, and it is considered to be a sensitive target for the treatment of breast cancer. A study involving 313 Chinese breast cancer patients found that the mutation frequency of AKT1 in Chinese breast cancer patients was 3.2%, and it is considered a sensitive target for the treatment of breast cancer [43]. HDAC2 is a poor prognostic factor in patients receiving anthracycline therapy and is positively correlated with breast cancer metastasis, progression, increased Ki-67, multidrug resistance protein, and negatively correlated with overall survival of patients [44]. The occurrence and development of breast cancer are closely related to the core proteins, which fully prove that the treatment of breast cancer by Chaihu-Shugan-San is the result of multimolecular, multitarget, and multipathway interaction.

The biological process of Gene Ontology (GO) involves the mRNA catabolic process, RNA catabolic process, telomere organization, nucleobase-containing compound catabolic process, heterocycle catabolic process, and so on. In cellular component, cytosolic part, focal adhesion, cell-substrate adherens junction, and cell-substrate junction are highly correlated with breast cancer. In the molecular function category, most proteins were addressed to ubiquitin-like protein ligase binding, protein domain specific binding, and Nop56p-associated pre-rRNA complex. In addition, the results of KEGG pathway analysis showed that the pathways mainly involved in apoptosis, cell cycle, transcriptional dysregulation, endocrine resistance, and viral infection. Estrogen receptor (ER) signal transduction pathway plays a central role in the development of breast cancer. ER can not only regulate the expression of certain genes through its mediated signal transduction pathway but also has extensive connections with many other signal transduction pathways, forming a signal transduction regulatory network [45]. ER binds to receptor proteins in the nucleus, and the receptor is activated. Activated ER-*α* and ER-*β* form homodimers or heterodimers. Some coregulators form complexes with dimers. The complexes bind to ER response elements to initiate transcription, thereby regulating the function of target genes, leading to abnormal cell proliferation and differentiation, and ultimately leading to tumorigenesis [46]. The increased mutation rate of ER-*α* in precancerous lesions of breast cancer affects the junction between ER-*α* zinc finger region and ligand binding domain, resulting in high sensitivity of the body to estrogen. Under the action of low levels of hormones, ER-*α* is highly bound to TNF-2 co-activator, which leads to the occurrence of tumors. For ER receptor-positive breast cancer patients, quantitative expression of ER receptor is an independent imaging factor to evaluate their prognosis, recurrence, and metastasis [47]. Abnormal activation of MAPK signal transduction pathway can lead to cell loss of apoptosis and differentiation ability, promote malignant transformation, abnormal proliferation, produce tumors, and further promote the proliferation of tumor cells. Therefore, inhibitors of some key kinases in the MAPK signaling pathway have become a hotspot in the treatment of breast cancer in recent years. Studies have found that Kruppel-like factor 4 [48] and pre-mRNA processing factor 4 [49] affect the growth, migration, and apoptosis of breast cancer cells through MAPK and are expected to become new targets for the treatment of breast cancer. Studies found that the activation or loss of FOXO function can inhibit the growth and metastasis of breast tumors [50], and the dysregulation of FOXO transcription factors has also become a key molecule in the endocrine resistance mechanism [51]. More and more attention has been paid to the relationship between viral infection and breast cancer. In particular, human papilloma virus (HPV) has a strong cause-and-effect relationship with breast cancer. Many studies have found that different HPV genotypes are associated with the prevalence of breast cancer and the nuclear prognosis. The relationship between viral infection and breast cancer has been paid more and more attention [52–54]. The relationship between Epstein–Barr Virus (EBV) and breast cancer has also been extensively studied, but the current evidence is less and more controversial [55]. It is proved again that Chaihu-Shugan-San treatment of breast cancer is through a combination of multiple biological pathways and multiple signaling pathways, but this multifeature is not only found in a single disease. Chaihu-Shugan-San is mainly involved in the regulation of neurotransmitters, regulation of inflammatory mediators of TRP channels, calcium signaling pathways, cyclic adenosine monophosphate signaling pathways, and neuroactive ligand-receptor interactions to play an antidepressant role [56]. Chaihu-Shugan-San can improve cognitive impairment in Alzheimer's disease through multitarget action, and its effect is verified by biological experiments [57]. These all embody the principle of “treating different diseases with the same treatment” in TCM.

## Figures and Tables

**Table 1:** Topological parameter of top 10 compounds.

ID	Molecule name	DC	BC	CC	ASPL
MOL000422	Kaempferol	317	0.011	0.377	2.651
MOL000354	Isorhamnetin	303	0.008	0.373	2.681
MOL000359	Sitosterol	233	0.005	0.360	2.774
MOL004328	Naringenin	199	0.058	0.397	2.520
MOL000358	Beta-sitosterol	139	0.004	0.357	2.802
MOL000098	Quercetin	116	0.009	0.377	2.654
MOL004609	Areapillin	101	0.008	0.372	2.690
MOL003044	Chrysoeriol	101	0.008	0.371	2.690
MOL000006	Luteolin	101	0.008	0.371	2.690
MOL004071	Hyndarin	101	0.063	0.374	2.677

**Table 2:** Topological parameter of top 10 core targets.

Target	DC	BC	CC	ASPL
HDAC1	1976	0.075	0.534	1.874
HTT	1695	0.094	0.526	1.900
AKT1	1136	0.031	0.495	2.019
HDGF	1095	0.021	0.499	2.002
RC3H1	930	0.026	0.501	1.996
CBX8	836	0.013	0.488	2.050
HDAC2	813	0.015	0.493	2.029
SUMO1	810	0.021	0.500	1.999
RPSA	761	0.013	0.489	2.047
RPLP0	736	0.011	0.481	2.081

## Data Availability

All data generated or analyzed during this study are included in this paper.

## Supplementary Materials

Supplementary Materials Table S1: active ingredients of Chaihu-Shugan-San. Table S2: topological parameters of Chaihu-Shugan-San targets. Table S3: the top 20 signal pathways of KEGG enrichment.
