# Supplementary material for: Potential Molecular Mechanisms of Chaihu-Shugan-San in Treatment of Breast Cancer Based on Network Pharmacology
Source: Evid Based Complement Alternat Med. 2020 Sep 25;2020:3670309. doi: 10.1155/2020/3670309 (PMC7533014; doi:10.1155/2020/3670309)
Supplement: Supplementary Materials — Table S1: active ingredients of Chaihu-Shugan-San. Table S2: topological parameters of Chaihu-Shugan-San targets. Table S3: the top 20 signal pathways of KEGG enrichment. [file 3670309.f1.zip › 3670309.f1/TableS1.docx]

Table S1 Active ingredient of Chaihu-Shugan-San

| Number | Molecule ID | Molecule name | OB% | DL | Herb name |
| --- | --- | --- | --- | --- | --- |
| 1 | MOL013381 | Marmin | 38.23 | 0.31 | Zhiqiao |
| 2 | MOL002341 | Hesperetin | 70.31 | 0.27 | Zhiqiao |
| 3 | MOL000358 | beta-sitosterol | 36.91 | 0.75 | Zhiqiao |
| 4 | MOL004328 | naringenin | 59.29 | 0.21 | Zhiqiao |
| 5 | MOL005828 | nobiletin | 61.67 | 0.52 | Zhiqiao |
|  |  |  |  |  |  |
| 1 | MOL001494 | Mandenol | 42 | 0.19 | Chuanxiong |
| 2 | MOL002135 | Myricanone | 40.6 | 0.51 | Chuanxiong |
| 3 | MOL002140 | Perlolyrine | 65.95 | 0.27 | Chuanxiong |
| 4 | MOL002151 | senkyunone | 47.66 | 0.24 | Chuanxiong |
| 5 | MOL002157 | wallichilide | 42.31 | 0.71 | Chuanxiong |
| 6 | MOL000359 | sitosterol | 36.91 | 0.75 | Chuanxiong |
| 7 | MOL000433 | FA | 68.96 | 0.71 | Chuanxiong |
|  |  |  |  |  |  |
| 1 | MOL001910 | 11alpha,12alpha-epoxy-3beta-23-dihydroxy-30-norolean-20-en-28,12beta-olide | 64.77 | 0.38 | Baishao |
| 2 | MOL001918 | paeoniflorgenone | 87.59 | 0.37 | Baishao |
| 3 | MOL001919 | (3S,5R,8R,9R,10S,14S)-3,17-dihydroxy-4,4,8,10,14-pentamethyl-2,3,5,6,7,9-hexahydro-1H-cyclopenta[a]phenanthrene-15,16-dione | 43.56 | 0.53 | Baishao |
| 4 | MOL001921 | Lactiflorin | 49.12 | 0.8 | Baishao |
| 5 | MOL001924 | paeoniflorin | 53.87 | 0.79 | Baishao |
| 6 | MOL001925 | paeoniflorin_qt | 68.18 | 0.4 | Baishao |
| 7 | MOL001928 | albiflorin_qt | 66.64 | 0.33 | Baishao |
| 8 | MOL001930 | benzoyl paeoniflorin | 31.27 | 0.75 | Baishao |
| 9 | MOL000211 | Mairin | 55.38 | 0.78 | Baishao |
| 10 | MOL000358 | beta-sitosterol | 36.91 | 0.75 | Baishao |
| 11 | MOL000359 | sitosterol | 36.91 | 0.75 | Baishao |
| 12 | MOL000422 | kaempferol | 41.88 | 0.24 | Baishao |
| 13 | MOL000492 | (+)-catechin | 54.83 | 0.24 | Baishao |
|  |  |  |  |  |  |
| 1 | MOL000359 | sitosterol | 36.91 | 0.75 | Chenpi |
| 2 | MOL004328 | naringenin | 59.29 | 0.21 | Chenpi |
| 3 | MOL005100 | 5,7-dihydroxy-2-(3-hydroxy-4-methoxyphenyl)chroman-4-one | 47.74 | 0.27 | Chenpi |
| 4 | MOL005815 | Citromitin | 86.9 | 0.51 | Chenpi |
| 5 | MOL005828 | nobiletin | 61.67 | 0.52 | Chenpi |
|  |  |  |  |  |  |
| 1 | MOL001645 | Linoleyl acetate | 42.1 | 0.2 | Chaihu |
| 2 | MOL002776 | Baicalin | 40.12 | 0.75 | Chaihu |
| 3 | MOL000449 | Stigmasterol | 43.83 | 0.76 | Chaihu |
| 4 | MOL000354 | isorhamnetin | 49.6 | 0.31 | Chaihu |
| 5 | MOL000422 | kaempferol | 41.88 | 0.24 | Chaihu |
| 6 | MOL004598 | 3,5,6,7-tetramethoxy-2-(3,4,5-trimethoxyphenyl)chromone | 31.97 | 0.59 | Chaihu |
| 7 | MOL004609 | Areapillin | 48.96 | 0.41 | Chaihu |
| 8 | MOL013187 | Cubebin | 57.13 | 0.64 | Chaihu |
| 9 | MOL004624 | Longikaurin A | 47.72 | 0.53 | Chaihu |
| 10 | MOL004628 | Octalupine | 47.82 | 0.28 | Chaihu |
| 11 | MOL004644 | Sainfuran | 79.91 | 0.23 | Chaihu |
| 12 | MOL004648 | Troxerutin | 31.6 | 0.28 | Chaihu |
| 13 | MOL004653 | (+)-Anomalin | 46.06 | 0.66 | Chaihu |
| 14 | MOL004702 | saikosaponin c_qt | 30.5 | 0.63 | Chaihu |
| 15 | MOL004718 | α-spinasterol | 42.98 | 0.76 | Chaihu |
| 16 | MOL000490 | petunidin | 30.05 | 0.31 | Chaihu |
| 17 | MOL000098 | quercetin | 46.43 | 0.28 | Chaihu |
|  |  |  |  |  |  |
| 1 | MOL003044 | Chryseriol | 35.85 | 0.27 | Xiangfu |
| 2 | MOL000354 | isorhamnetin | 49.6 | 0.31 | Xiangfu |
| 3 | MOL003542 | 8-Isopentenyl-kaempferol | 38.04 | 0.39 | Xiangfu |
| 4 | MOL000358 | beta-sitosterol | 36.91 | 0.75 | Xiangfu |
| 5 | MOL000359 | sitosterol | 36.91 | 0.75 | Xiangfu |
| 6 | MOL004027 | 1,4-Epoxy-16-hydroxyheneicos-1,3,12,14,18-pentaene | 45.1 | 0.24 | Xiangfu |
| 7 | MOL004053 | Isodalbergin | 35.45 | 0.2 | Xiangfu |
| 8 | MOL004058 | Khell | 33.19 | 0.19 | Xiangfu |
| 9 | MOL004059 | khellol glucoside | 74.96 | 0.72 | Xiangfu |
| 10 | MOL010489 | Resivit | 30.84 | 0.27 | Xiangfu |
| 11 | MOL004068 | rosenonolactone | 79.84 | 0.37 | Xiangfu |
| 12 | MOL004071 | Hyndarin | 73.94 | 0.64 | Xiangfu |
| 13 | MOL004074 | stigmasterol glucoside_qt | 43.83 | 0.76 | Xiangfu |
| 14 | MOL004077 | sugeonyl acetate | 45.08 | 0.2 | Xiangfu |
| 15 | MOL000422 | kaempferol | 41.88 | 0.24 | Xiangfu |
| 16 | MOL000449 | Stigmasterol | 43.83 | 0.76 | Xiangfu |
| 17 | MOL000006 | luteolin | 36.16 | 0.25 | Xiangfu |
| 18 | MOL000098 | quercetin | 46.43 | 0.28 | Xiangfu |
|  |  |  |  |  |  |
| 1 | MOL001484 | Inermine | 75.18 | 0.54 | Gancao |
| 2 | MOL001792 | DFV | 32.76 | 0.18 | Gancao |
| 3 | MOL004806 | euchrenone | 30.29 | 0.57 | Gancao |
| 4 | MOL004810 | glyasperin F | 75.84 | 0.54 | Gancao |
| 5 | MOL004829 | Glepidotin B | 64.46 | 0.34 | Gancao |
| 6 | MOL004860 | licorice glycoside E | 32.89 | 0.27 | Gancao |
| 7 | MOL004904 | licopyranocoumarin | 80.36 | 0.65 | Gancao |
| 8 | MOL004988 | Kanzonol F | 32.47 | 0.89 | Gancao |
| 9 | MOL004989 | 6-prenylated eriodictyol | 39.22 | 0.41 | Gancao |
| 10 | MOL004993 | 8-prenylated eriodictyol | 53.79 | 0.4 | Gancao |
| 11 | MOL005007 | Glyasperins M | 72.67 | 0.59 | Gancao |
| 12 | MOL005008 | Glycyrrhiza flavonol A | 41.28 | 0.6 | Gancao |
| 13 | MOL005013 | 18α-hydroxyglycyrrhetic acid | 41.16 | 0.71 | Gancao |
| 14 | MOL005018 | Xambioona | 54.85 | 0.87 | Gancao |
| 15 | MOL005020 | dehydroglyasperins C | 53.82 | 0.37 | Gancao |
| 16 | MOL000211 | Mairin | 55.38 | 0.78 | Gancao |
| 17 | MOL002311 | Glycyrol | 90.78 | 0.67 | Gancao |
| 18 | MOL000239 | Jaranol | 50.83 | 0.29 | Gancao |
| 19 | MOL002565 | Medicarpin | 49.22 | 0.34 | Gancao |
| 20 | MOL000354 | isorhamnetin | 49.6 | 0.31 | Gancao |
| 21 | MOL000359 | sitosterol | 36.91 | 0.75 | Gancao |
| 22 | MOL003656 | Lupiwighteone | 51.64 | 0.37 | Gancao |
| 23 | MOL003896 | 7-Methoxy-2-methyl isoflavone | 42.56 | 0.2 | Gancao |
| 24 | MOL000392 | formononetin | 69.67 | 0.21 | Gancao |
| 25 | MOL000417 | Calycosin | 47.75 | 0.24 | Gancao |
| 26 | MOL000422 | kaempferol | 41.88 | 0.24 | Gancao |
| 27 | MOL004328 | naringenin | 59.29 | 0.21 | Gancao |
| 28 | MOL004805 | (2S)-2-[4-hydroxy-3-(3-methylbut-2-enyl)phenyl]-8,8-dimethyl-2,3-dihydropyrano[2,3-f]chromen-4-one | 31.79 | 0.72 | Gancao |
| 29 | MOL004808 | glyasperin B | 65.22 | 0.44 | Gancao |
| 30 | MOL004811 | Glyasperin C | 45.56 | 0.4 | Gancao |
| 31 | MOL004814 | Isotrifoliol | 31.94 | 0.42 | Gancao |
| 32 | MOL004815 | (E)-1-(2,4-dihydroxyphenyl)-3-(2,2-dimethylchromen-6-yl)prop-2-en-1-one | 39.62 | 0.35 | Gancao |
| 33 | MOL004820 | kanzonols W | 50.48 | 0.52 | Gancao |
| 34 | MOL004824 | (2S)-6-(2,4-dihydroxyphenyl)-2-(2-hydroxypropan-2-yl)-4-methoxy-2,3-dihydrofuro[3,2-g]chromen-7-one | 60.25 | 0.63 | Gancao |
| 35 | MOL004827 | Semilicoisoflavone B | 48.78 | 0.55 | Gancao |
| 36 | MOL004828 | Glepidotin A | 44.72 | 0.35 | Gancao |
| 37 | MOL004833 | Phaseolinisoflavan | 32.01 | 0.45 | Gancao |
| 38 | MOL004835 | Glypallichalcone | 61.6 | 0.19 | Gancao |
| 39 | MOL004838 | 8-(6-hydroxy-2-benzofuranyl)-2,2-dimethyl-5-chromenol | 58.44 | 0.38 | Gancao |
| 40 | MOL004841 | Licochalcone B | 76.76 | 0.19 | Gancao |
| 41 | MOL004848 | licochalcone G | 49.25 | 0.32 | Gancao |
| 42 | MOL004849 | 3-(2,4-dihydroxyphenyl)-8-(1,1-dimethylprop-2-enyl)-7-hydroxy-5-methoxy-coumarin | 59.62 | 0.43 | Gancao |
| 43 | MOL004855 | Licoricone | 63.58 | 0.47 | Gancao |
| 44 | MOL004856 | Gancaonin A | 51.08 | 0.4 | Gancao |
| 45 | MOL004857 | Gancaonin B | 48.79 | 0.45 | Gancao |
| 46 | MOL004863 | 3-(3,4-dihydroxyphenyl)-5,7-dihydroxy-8-(3-methylbut-2-enyl)chromone | 66.37 | 0.41 | Gancao |
| 47 | MOL004864 | 5,7-dihydroxy-3-(4-methoxyphenyl)-8-(3-methylbut-2-enyl)chromone | 30.49 | 0.41 | Gancao |
| 48 | MOL004866 | 2-(3,4-dihydroxyphenyl)-5,7-dihydroxy-6-(3-methylbut-2-enyl)chromone | 44.15 | 0.41 | Gancao |
| 49 | MOL004879 | Glycyrin | 52.61 | 0.47 | Gancao |
| 50 | MOL004882 | Licocoumarone | 33.21 | 0.36 | Gancao |
| 51 | MOL004883 | Licoisoflavone | 41.61 | 0.42 | Gancao |
| 52 | MOL004884 | Licoisoflavone B | 38.93 | 0.55 | Gancao |
| 53 | MOL004885 | licoisoflavanone | 52.47 | 0.54 | Gancao |
| 54 | MOL004891 | shinpterocarpin | 80.3 | 0.73 | Gancao |
| 55 | MOL004898 | (E)-3-[3,4-dihydroxy-5-(3-methylbut-2-enyl)phenyl]-1-(2,4-dihydroxyphenyl)prop-2-en-1-one | 46.27 | 0.31 | Gancao |
| 56 | MOL004903 | liquiritin | 65.69 | 0.74 | Gancao |
| 57 | MOL004905 | 3,22-Dihydroxy-11-oxo-delta(12)-oleanene-27-alpha-methoxycarbonyl-29-oic acid | 34.32 | 0.55 | Gancao |
| 58 | MOL004907 | Glyzaglabrin | 61.07 | 0.35 | Gancao |
| 59 | MOL004908 | Glabridin | 53.25 | 0.47 | Gancao |
| 60 | MOL004910 | Glabranin | 52.9 | 0.31 | Gancao |
| 61 | MOL004911 | Glabrene | 46.27 | 0.44 | Gancao |
| 62 | MOL004912 | Glabrone | 52.51 | 0.5 | Gancao |
| 63 | MOL004913 | 1,3-dihydroxy-9-methoxy-6-benzofurano[3,2-c]chromenone | 48.14 | 0.43 | Gancao |
| 64 | MOL004914 | 1,3-dihydroxy-8,9-dimethoxy-6-benzofurano[3,2-c]chromenone | 62.9 | 0.53 | Gancao |
| 65 | MOL004915 | Eurycarpin A | 43.28 | 0.37 | Gancao |
| 66 | MOL004917 | glycyroside | 37.25 | 0.79 | Gancao |
| 67 | MOL004924 | (-)-Medicocarpin | 40.99 | 0.95 | Gancao |
| 68 | MOL004935 | Sigmoidin-B | 34.88 | 0.41 | Gancao |
| 69 | MOL004941 | (2R)-7-hydroxy-2-(4-hydroxyphenyl)chroman-4-one | 71.12 | 0.18 | Gancao |
| 70 | MOL004945 | (2S)-7-hydroxy-2-(4-hydroxyphenyl)-8-(3-methylbut-2-enyl)chroman-4-one | 36.57 | 0.32 | Gancao |
| 71 | MOL004948 | Isoglycyrol | 44.7 | 0.84 | Gancao |
| 72 | MOL004949 | Isolicoflavonol | 45.17 | 0.42 | Gancao |
| 73 | MOL004957 | HMO | 38.37 | 0.21 | Gancao |
| 74 | MOL004959 | 1-Methoxyphaseollidin | 69.98 | 0.64 | Gancao |
| 75 | MOL004961 | Quercetin der. | 46.45 | 0.33 | Gancao |
| 76 | MOL004966 | 3'-Hydroxy-4'-O-Methylglabridin | 43.71 | 0.57 | Gancao |
| 77 | MOL000497 | licochalcone a | 40.79 | 0.29 | Gancao |
| 78 | MOL004974 | 3'-Methoxyglabridin | 46.16 | 0.57 | Gancao |
| 79 | MOL004978 | 2-[(3R)-8,8-dimethyl-3,4-dihydro-2H-pyrano[6,5-f]chromen-3-yl]-5-methoxyphenol | 36.21 | 0.52 | Gancao |
| 80 | MOL004980 | Inflacoumarin A | 39.71 | 0.33 | Gancao |
| 81 | MOL004985 | icos-5-enoic acid | 30.7 | 0.2 | Gancao |
| 82 | MOL004990 | 7,2',4'-trihydroxy－5-methoxy-3－arylcoumarin | 83.71 | 0.27 | Gancao |
| 83 | MOL004991 | 7-Acetoxy-2-methylisoflavone | 38.92 | 0.26 | Gancao |
| 84 | MOL004996 | gadelaidic acid | 30.7 | 0.2 | Gancao |
| 85 | MOL000500 | Vestitol | 74.66 | 0.21 | Gancao |
| 86 | MOL005000 | Gancaonin G | 60.44 | 0.39 | Gancao |
| 87 | MOL005001 | Gancaonin H | 50.1 | 0.78 | Gancao |
| 88 | MOL005003 | Licoagrocarpin | 58.81 | 0.58 | Gancao |
| 89 | MOL005012 | Licoagroisoflavone | 57.28 | 0.49 | Gancao |
| 90 | MOL005016 | Odoratin | 49.95 | 0.3 | Gancao |
| 91 | MOL005017 | Phaseol | 78.77 | 0.58 | Gancao |
| 92 | MOL000098 | quercetin | 46.43 | 0.28 | Gancao |
