# Supplementary material for: Potential Molecular Mechanisms of Chaihu-Shugan-San in Treatment of Breast Cancer Based on Network Pharmacology
Source: Evid Based Complement Alternat Med. 2020 Sep 25;2020:3670309. doi: 10.1155/2020/3670309 (PMC7533014; doi:10.1155/2020/3670309)
Supplement: Supplementary Materials — Table S1: active ingredients of Chaihu-Shugan-San. Table S2: topological parameters of Chaihu-Shugan-San targets. Table S3: the top 20 signal pathways of KEGG enrichment. [file 3670309.f1.zip › 3670309.f1/TableS2.docx]

Table S2 Topological parameters of Chaihu-Shugan-San targets

| Target | DC | BC | CC | ASPL |
| --- | --- | --- | --- | --- |
| HDAC1 | 1976 | 0.07503191 | 0.53375539 | 1.87351738 |
| HTT | 1695 | 0.09375648 | 0.52640078 | 1.89969325 |
| AKT1 | 1136 | 0.03081987 | 0.49539054 | 2.01860941 |
| HDGF | 1095 | 0.01952154 | 0.49941276 | 2.00235174 |
| RC3H1 | 930 | 0.02616143 | 0.5009476 | 1.99621677 |
| CBX8 | 836 | 0.0128786 | 0.4877319 | 2.05030675 |
| HDAC2 | 813 | 0.01548696 | 0.49294355 | 2.02862986 |
| SUMO1 | 810 | 0.02099322 | 0.50028134 | 1.99887526 |
| RPSA | 761 | 0.01284078 | 0.48851149 | 2.04703476 |
| RPLP0 | 736 | 0.01093901 | 0.48058968 | 2.0807771 |
| LRRK2 | 717 | 0.01825961 | 0.48865794 | 2.04642127 |
| RPL18 | 712 | 0.00748745 | 0.47023752 | 2.12658487 |
| UBB | 701 | 0.00804147 | 0.4808496 | 2.07965235 |
| UBE2D1 | 687 | 0.00946783 | 0.47544968 | 2.10327198 |
| UBE2D2 | 665 | 0.00492539 | 0.46164739 | 2.16615542 |
| RPL23A | 653 | 0.0125799 | 0.48729447 | 2.05214724 |
| RPL24 | 635 | 0.00360405 | 0.47225844 | 2.11748466 |
| UBE2D3 | 634 | 0.00565806 | 0.47026013 | 2.12648262 |
| UBE2I | 622 | 0.00394507 | 0.45911182 | 2.17811861 |
| KDM1A | 609 | 0.00901458 | 0.4894405 | 2.04314928 |
| RPA2 | 603 | 0.01061459 | 0.48106247 | 2.07873211 |
| TUFM | 599 | 0.00325571 | 0.45969448 | 2.17535787 |
| RPL3 | 574 | 0.00589428 | 0.48377523 | 2.06707566 |
| IQCB1 | 562 | 0.00682985 | 0.47367656 | 2.11114519 |
| RPL4 | 556 | 0.00862169 | 0.45361781 | 2.20449898 |
| TUBG1 | 550 | 0.00473042 | 0.46095112 | 2.1694274 |
| RPL7 | 542 | 0.00747438 | 0.45530726 | 2.19631902 |
| HIST1H2BD | 536 | 0.00395343 | 0.46112499 | 2.16860941 |
| RPL6 | 531 | 0.00959448 | 0.46944751 | 2.1301636 |
| RPL9 | 516 | 0.00297817 | 0.46073397 | 2.1704499 |
| RPL5 | 514 | 0.0072524 | 0.46859278 | 2.13404908 |
| RPL7A | 509 | 0.00395811 | 0.46390286 | 2.15562372 |
| RPL10 | 496 | 0.00564982 | 0.47273782 | 2.11533742 |
| SH3KBP1 | 489 | 0.00345635 | 0.46249882 | 2.16216769 |
| RPL8 | 485 | 0.00273989 | 0.4459441 | 2.24243354 |
| RPL13 | 481 | 0.00408632 | 0.47059956 | 2.12494888 |
| RPL11 | 459 | 0.00290271 | 0.45767233 | 2.18496933 |
| BAG6 | 455 | 0.00728001 | 0.47057691 | 2.12505112 |
| ISG15 | 447 | 0.00861629 | 0.47337851 | 2.11247444 |
| IKBKE | 446 | 0.00474975 | 0.4658252 | 2.14672802 |
| KPNA2 | 445 | 0.00212141 | 0.454039 | 2.20245399 |
| KPNB1 | 444 | 0.00757271 | 0.47445787 | 2.10766871 |
| RPA3 | 443 | 0.00123577 | 0.44637152 | 2.2402863 |
| ADRB2 | 443 | 0.00123577 | 0.44637152 | 2.2402863 |
| RPA1 | 443 | 0.00123577 | 0.44637152 | 2.2402863 |
| TUBA4A | 443 | 0.00123577 | 0.44637152 | 2.2402863 |
| CCT6A | 443 | 0.00123577 | 0.44637152 | 2.2402863 |
| MDC1 | 443 | 0.00123577 | 0.44637152 | 2.2402863 |
| CBL | 443 | 0.00123577 | 0.44637152 | 2.2402863 |
| PAXIP1 | 443 | 0.00123577 | 0.44637152 | 2.2402863 |
| NCOR1 | 443 | 0.00123577 | 0.44637152 | 2.2402863 |
| RAB7A | 443 | 0.00123577 | 0.44637152 | 2.2402863 |
| SMURF1 | 443 | 0.00318927 | 0.46553694 | 2.14805726 |
| USP7 | 441 | 0.00394458 | 0.46418909 | 2.15429448 |
| RNF2 | 436 | 0.0047906 | 0.46075568 | 2.17034765 |
| PARP1 | 431 | 0.00275388 | 0.46082081 | 2.1700409 |
| NINL | 424 | 0.00480583 | 0.46904225 | 2.13200409 |
| ABCE1 | 417 | 0.00333786 | 0.4607991 | 2.17014315 |
| MRPL58 | 407 | 0.00222345 | 0.44979994 | 2.22321063 |
| CAV1 | 406 | 0.00453059 | 0.47992934 | 2.08364008 |
| TMEM216 | 401 | 0.00463745 | 0.46077739 | 2.1702454 |
| SNW1 | 391 | 0.00335291 | 0.46112499 | 2.16860941 |
| TUBA1A | 383 | 0.00367189 | 0.46527117 | 2.14928425 |
| CAPZA2 | 375 | 0.0020552 | 0.44622895 | 2.24100204 |
| ABL1 | 374 | 0.00265841 | 0.4427944 | 2.25838446 |
| CASP8 | 374 | 0.00156705 | 0.44345697 | 2.25501022 |
| CASP3 | 374 | 0.00430488 | 0.44089803 | 2.26809816 |
| ICAM1 | 374 | 0.00193588 | 0.45011046 | 2.22167689 |
| HSP90AB1 | 373 | 0.00156292 | 0.43259023 | 2.31165644 |
| HSP90AA1 | 372 | 0.00408047 | 0.44673853 | 2.23844581 |
| CANX | 372 | 0.00178131 | 0.43304995 | 2.30920245 |
| HSPD1 | 372 | 0.00178131 | 0.43304995 | 2.30920245 |
| BAG3 | 370 | 0.00464481 | 0.46105978 | 2.16891616 |
| TUBA1C | 368 | 0.00365733 | 0.46319977 | 2.15889571 |
| HSPA1A | 365 | 0.00285744 | 0.45488372 | 2.19836401 |
| PCM1 | 360 | 0.00494298 | 0.43660714 | 2.29038855 |
| HSPA1B | 357 | 0.00455824 | 0.47964689 | 2.08486708 |
| PCNA | 356 | 0.00428247 | 0.44576117 | 2.24335378 |
| HSPA4 | 353 | 0.00447046 | 0.46047366 | 2.17167689 |
| HSPB1 | 351 | 0.00636654 | 0.46591396 | 2.14631902 |
| HSPA5 | 351 | 0.00139651 | 0.44996549 | 2.22239264 |
| JUN | 348 | 0.00131182 | 0.42819615 | 2.33537832 |
| HSPA9 | 346 | 0.00425114 | 0.45825134 | 2.18220859 |
| HSPA8 | 342 | 0.00286859 | 0.43764264 | 2.28496933 |
| MATR3 | 340 | 0.00498054 | 0.46611381 | 2.14539877 |
| ITGA4 | 340 | 0.00319951 | 0.44396023 | 2.25245399 |
| PRMT1 | 338 | 0.00268943 | 0.44682018 | 2.23803681 |
| TSG101 | 334 | 0.00364472 | 0.45069124 | 2.21881391 |
| LNX1 | 334 | 0.00312474 | 0.45389149 | 2.20316973 |
| PCBP1 | 327 | 0.00211024 | 0.45696664 | 2.18834356 |
| DNAJA1 | 325 | 0.00659617 | 0.43670462 | 2.2898773 |
| TRAF1 | 325 | 0.00364743 | 0.45558299 | 2.19498978 |
| RPL23 | 323 | 0.0025333 | 0.42495872 | 2.35316973 |
| TRAF2 | 319 | 0.0012423 | 0.43146424 | 2.31768916 |
| TRAF6 | 319 | 0.00172471 | 0.42992791 | 2.32597137 |
| PARK2 | 318 | 0.00293947 | 0.44586278 | 2.24284254 |
| FAF2 | 317 | 0.00345433 | 0.45486256 | 2.19846626 |
| CCT3 | 315 | 0.00328883 | 0.47180279 | 2.11952965 |
| TRIP6 | 311 | 0.00550209 | 0.45577407 | 2.19406953 |
| EIF4A3 | 311 | 0.00211029 | 0.42555043 | 2.34989775 |
| MCM5 | 310 | 0.00100785 | 0.45277778 | 2.20858896 |
| SFN | 310 | 0.00418684 | 0.45401792 | 2.20255624 |
| MCM7 | 309 | 0.00181768 | 0.43734907 | 2.28650307 |
| HECW2 | 308 | 0.00183427 | 0.41744921 | 2.39550102 |
| CEP250 | 306 | 0.00329071 | 0.40119785 | 2.49253579 |
| TOP1 | 306 | 0.00258018 | 0.45477796 | 2.19887526 |
| MCC | 304 | 0.00359669 | 0.45726576 | 2.18691207 |
| GOLGA2 | 302 | 9.27E-04 | 0.44973788 | 2.22351738 |
| MCM2 | 302 | 0.00141909 | 0.4524845 | 2.21002045 |
| LZTS2 | 301 | 0.00319021 | 0.45780087 | 2.18435583 |
| BARD1 | 300 | 0.00500137 | 0.44513222 | 2.24652352 |
| TNFRSF1A | 300 | 0.00331151 | 0.45347058 | 2.20521472 |
| CEP128 | 299 | 0.00335583 | 0.43996581 | 2.27290389 |
| VAPA | 299 | 0.00190905 | 0.45685991 | 2.18885481 |
| CDC37 | 298 | 0.002231 | 0.45605036 | 2.19274029 |
| MAGOH | 297 | 0.00225452 | 0.44576117 | 2.24335378 |
| SMAD3 | 287 | 0.00233474 | 0.43287744 | 2.3101227 |
| SMAD1 | 287 | 0.00122759 | 0.44303511 | 2.25715746 |
| MED4 | 287 | 0.00121929 | 0.4397482 | 2.27402863 |
| SMAD4 | 285 | 0.00228001 | 0.45087824 | 2.21789366 |
| CTNNB1 | 283 | 0.0021734 | 0.44315556 | 2.25654397 |
| SMAD2 | 283 | 0.00239763 | 0.43776017 | 2.28435583 |
| PTEN | 282 | 0.00228464 | 0.45273586 | 2.20879346 |
| AURKB | 274 | 0.00328686 | 0.44688143 | 2.23773006 |
| PSMC5 | 273 | 0.0018149 | 0.43322259 | 2.30828221 |
| LYN | 273 | 0.00185633 | 0.45844466 | 2.18128834 |
| PSMD2 | 273 | 0.00462254 | 0.45073279 | 2.21860941 |
| PSMC3 | 272 | 0.00269838 | 0.45416551 | 2.20184049 |
| WDR5 | 272 | 0.00285913 | 0.44038184 | 2.27075665 |
| PSMD4 | 270 | 0.00448985 | 0.44177432 | 2.26359918 |
| TGFBR1 | 270 | 0.00261838 | 0.45393363 | 2.20296524 |
| CSNK2A2 | 269 | 0.00170987 | 0.44515248 | 2.24642127 |
| PSMA3 | 269 | 0.00123494 | 0.44806891 | 2.23179959 |
| PSMA1 | 269 | 0.00184426 | 0.46910975 | 2.13169734 |
| CSNK2B | 268 | 0.0020643 | 0.44319572 | 2.25633947 |
| UBC | 268 | 0.00123921 | 0.43114089 | 2.3194274 |
| MEPCE | 266 | 0.00127816 | 0.4239088 | 2.35899796 |
| HGS | 265 | 0.00101132 | 0.41491663 | 2.4101227 |
| ITCH | 265 | 0.00101132 | 0.41491663 | 2.4101227 |
| TERF1 | 265 | 0.00101132 | 0.41491663 | 2.4101227 |
| TERF2 | 264 | 0.00296541 | 0.44377893 | 2.25337423 |
| RUVBL2 | 263 | 0.00219291 | 0.45075356 | 2.21850716 |
| MAPK14 | 263 | 0.00119543 | 0.45654001 | 2.19038855 |
| GAPDH | 262 | 0.00474317 | 0.43827022 | 2.28169734 |
| LMNA | 261 | 0.00251735 | 0.4379562 | 2.28333333 |
| CRK | 261 | 0.00246341 | 0.43195972 | 2.31503067 |
| CAND1 | 260 | 0.00199916 | 0.45321841 | 2.20644172 |
| TCP1 | 260 | 0.00204694 | 0.45713752 | 2.18752556 |
| PRKDC | 259 | 5.68E-04 | 0.42881571 | 2.33200409 |
| UCHL5 | 259 | 0.00106025 | 0.43674363 | 2.2896728 |
| ILF2 | 258 | 0.00234882 | 0.44484876 | 2.24795501 |
| MAPK3 | 258 | 0.00220524 | 0.44175437 | 2.26370143 |
| CREBBP | 258 | 0.00153686 | 0.44920081 | 2.22617587 |
| ATF2 | 257 | 0.00270573 | 0.43613985 | 2.29284254 |
| ILK | 257 | 9.28E-04 | 0.43462803 | 2.300818 |
| MAPK1 | 257 | 0.00156952 | 0.43462803 | 2.300818 |
| ILF3 | 256 | 0.00148913 | 0.4024857 | 2.48456033 |
| TCF4 | 255 | 0.00141901 | 0.44036202 | 2.2708589 |
| MAPK8 | 255 | 0.00296144 | 0.44808943 | 2.23169734 |
| U2AF2 | 255 | 0.00189674 | 0.42326668 | 2.36257669 |
| PRKCD | 254 | 0.00144463 | 0.44069935 | 2.26912065 |
| PRKCA | 254 | 0.00182479 | 0.40687274 | 2.45777096 |
| SSX2IP | 253 | 0.00234403 | 0.43030623 | 2.32392638 |
| MED23 | 253 | 0.00174296 | 0.44965517 | 2.22392638 |
| GSK3B | 252 | 0.00183479 | 0.43848637 | 2.2805726 |
| PRKACA | 252 | 0.00217904 | 0.4456596 | 2.24386503 |
| EED | 251 | 0.00257414 | 0.43522763 | 2.29764826 |
| AP2M1 | 251 | 0.00263217 | 0.44688143 | 2.23773006 |
| CCDC8 | 250 | 0.00269156 | 0.44649379 | 2.2396728 |
| PRKAB1 | 250 | 0.00100393 | 0.4500069 | 2.22218814 |
| POU5F1P4 | 249 | 0.00276574 | 0.42804622 | 2.33619632 |
| NR3C1 | 249 | 4.70E-04 | 0.45008974 | 2.22177914 |
| UBQLN1 | 249 | 4.70E-04 | 0.45008974 | 2.22177914 |
| SUV39H1 | 249 | 4.70E-04 | 0.45008974 | 2.22177914 |
| CHUK | 249 | 4.70E-04 | 0.45008974 | 2.22177914 |
| GRB2 | 249 | 4.70E-04 | 0.45008974 | 2.22177914 |
| IKBKB | 249 | 4.70E-04 | 0.45008974 | 2.22177914 |
| GRK5 | 249 | 4.70E-04 | 0.45008974 | 2.22177914 |
| CHD3 | 249 | 4.70E-04 | 0.45008974 | 2.22177914 |
| PRPF40A | 249 | 4.70E-04 | 0.45008974 | 2.22177914 |
| SRC | 249 | 4.70E-04 | 0.45008974 | 2.22177914 |
| SPTAN1 | 249 | 4.70E-04 | 0.45008974 | 2.22177914 |
| DYNLL1 | 249 | 4.70E-04 | 0.45008974 | 2.22177914 |
| IFI16 | 249 | 4.70E-04 | 0.45008974 | 2.22177914 |
| FN1 | 249 | 4.70E-04 | 0.45008974 | 2.22177914 |
| PABPC1 | 248 | 8.62E-04 | 0.42840247 | 2.33425358 |
| CFTR | 248 | 7.76E-04 | 0.44331626 | 2.25572597 |
| RUVBL1 | 247 | 0.00171851 | 0.46075568 | 2.17034765 |
| FOS | 246 | 0.00170216 | 0.43194064 | 2.31513292 |
| POU5F1 | 246 | 8.27E-04 | 0.44171447 | 2.26390593 |
| FLNA | 244 | 0.00135874 | 0.4521707 | 2.21155419 |
| SOX2 | 244 | 0.00150362 | 0.44753581 | 2.23445808 |
| KHDRBS1 | 243 | 0.00166169 | 0.44596443 | 2.24233129 |
| SP1 | 242 | 0.00162819 | 0.43155944 | 2.31717791 |
| CDK9 | 242 | 0.00103624 | 0.4301359 | 2.32484663 |
| CDKN1A | 241 | 0.00184954 | 0.44774069 | 2.23343558 |
| CDKN2A | 241 | 4.54E-04 | 0.43377983 | 2.30531697 |
| POLR2A | 241 | 0.00139492 | 0.44089803 | 2.26809816 |
| HIST1H4D | 240 | 9.52E-04 | 0.42238922 | 2.36748466 |
| HIST1H4J | 239 | 0.00141894 | 0.44173442 | 2.26380368 |
| HIST1H4F | 239 | 0.00133972 | 0.42051855 | 2.37801636 |
| CDK4 | 239 | 0.00129637 | 0.42945593 | 2.32852761 |
| AHSA1 | 238 | 0.00118898 | 0.45698799 | 2.18824131 |
| CDK2 | 238 | 0.00118898 | 0.45698799 | 2.18824131 |
| EIF6 | 238 | 0.0018733 | 0.44245385 | 2.2601227 |
| SUMO2 | 238 | 0.00144402 | 0.42269957 | 2.36574642 |
| IKBKG | 237 | 8.11E-04 | 0.4265899 | 2.34417178 |
| PML | 235 | 0.00213821 | 0.46374887 | 2.15633947 |
| SNCA | 235 | 0.00109222 | 0.40816327 | 2.45 |
| HNRNPU | 235 | 0.00150811 | 0.44038184 | 2.27075665 |
| LGR4 | 234 | 0.00285457 | 0.43335697 | 2.30756646 |
| HIST1H2BE | 232 | 0.00211922 | 0.43209331 | 2.31431493 |
| HIST1H3D | 232 | 9.53E-04 | 0.43268593 | 2.31114519 |
| SMARCA4 | 231 | 4.96E-04 | 0.43358752 | 2.30633947 |
| HIST1H3A | 231 | 0.00131054 | 0.45956487 | 2.17597137 |
| PLK1 | 231 | 0.00229981 | 0.46309011 | 2.15940695 |
| HIST1H3G | 231 | 4.44E-04 | 0.41035539 | 2.43691207 |
| NANOG | 231 | 1.00E-03 | 0.41950843 | 2.38374233 |
| HIST1H3I | 231 | 5.69E-04 | 0.44639189 | 2.24018405 |
| HIST1H3E | 229 | 0.00151638 | 0.44452525 | 2.249591 |
| SMN2 | 227 | 0.00133384 | 0.4265899 | 2.34417178 |
| HIST1H3C | 227 | 0.00197466 | 0.43911638 | 2.27730061 |
| HIST1H4A | 226 | 0.00139641 | 0.43416496 | 2.30327198 |
| HIST1H3B | 226 | 0.00133736 | 0.46164739 | 2.16615542 |
| CDC42 | 226 | 7.74E-04 | 0.44357765 | 2.25439673 |
| HIST1H3J | 226 | 5.50E-04 | 0.44553779 | 2.24447853 |
| SMN1 | 225 | 0.00142058 | 0.44618824 | 2.24120654 |
| HIST1H4K | 225 | 0.00182884 | 0.43976798 | 2.27392638 |
| HIST1H3H | 224 | 0.00226251 | 0.44788423 | 2.23271984 |
| CD81 | 223 | 0.00280183 | 0.42994681 | 2.32586912 |
| HIST1H2BG | 223 | 0.00209199 | 0.41125268 | 2.43159509 |
| HIST1H2BF | 223 | 0.00101513 | 0.41620563 | 2.40265849 |
| PLCG1 | 223 | 7.25E-04 | 0.45096141 | 2.21748466 |
| CDK1 | 222 | 8.60E-04 | 0.42166077 | 2.37157464 |
| HIST1H2BC | 222 | 8.60E-04 | 0.42166077 | 2.37157464 |
| CDC5L | 222 | 8.00E-04 | 0.42949365 | 2.32832311 |
| SF3A1 | 221 | 6.01E-04 | 0.41295444 | 2.42157464 |
| FBXW7 | 221 | 0.00193229 | 0.44913892 | 2.22648262 |
| HIST1H2BI | 221 | 0.00163858 | 0.42308358 | 2.36359918 |
| PIN1 | 221 | 0.00183435 | 0.42178807 | 2.3708589 |
| FBXO25 | 221 | 8.51E-04 | 0.43994602 | 2.27300613 |
| YWHAQ | 220 | 5.96E-04 | 0.41291957 | 2.42177914 |
| COPS6 | 220 | 5.96E-04 | 0.41291957 | 2.42177914 |
| FBXO6 | 220 | 5.96E-04 | 0.41291957 | 2.42177914 |
| COPS5 | 220 | 5.96E-04 | 0.41291957 | 2.42177914 |
| PKM | 220 | 5.96E-04 | 0.41291957 | 2.42177914 |
| XRCC6 | 219 | 0.00125233 | 0.44349719 | 2.25480573 |
| PIK3R1 | 218 | 0.00114857 | 0.42341328 | 2.36175869 |
| STIP1 | 217 | 0.00132404 | 0.42189724 | 2.3702454 |
| FUS | 217 | 5.29E-04 | 0.41990468 | 2.38149284 |
| TCTN3 | 216 | 8.66E-04 | 0.40230358 | 2.48568507 |
| RNPS1 | 215 | 0.00269778 | 0.43304995 | 2.30920245 |
| FYN | 215 | 0.00108811 | 0.40665281 | 2.4591002 |
| IGSF8 | 215 | 9.00E-04 | 0.41556896 | 2.40633947 |
| SKP1 | 215 | 0.00180744 | 0.43686068 | 2.2890593 |
| SET | 215 | 5.73E-04 | 0.43891931 | 2.27832311 |
| SFPQ | 214 | 0.00160707 | 0.38569231 | 2.59274029 |
| YBX1 | 214 | 9.33E-04 | 0.42748492 | 2.3392638 |
| SRSF1 | 214 | 8.58E-04 | 0.4317309 | 2.31625767 |
| OTUB1 | 213 | 0.00124707 | 0.42144273 | 2.37280164 |
| NTRK1 | 213 | 0.00133501 | 0.43447357 | 2.30163599 |
| ATP5A1 | 211 | 9.67E-04 | 0.43567356 | 2.29529652 |
| NPM1 | 210 | 0.00108688 | 0.44143534 | 2.26533742 |
| EIF1B | 210 | 0.0010912 | 0.43106488 | 2.3198364 |
| ATM | 210 | 0.00153137 | 0.43986687 | 2.27341513 |
| EZH2 | 209 | 3.18E-04 | 0.40673737 | 2.45858896 |
| HIST1H3F | 209 | 6.65E-04 | 0.42653408 | 2.34447853 |
| BTRC | 208 | 0.00179458 | 0.41821681 | 2.39110429 |
| STAT3 | 208 | 8.93E-04 | 0.42645969 | 2.34488753 |
| STAU1 | 207 | 5.87E-04 | 0.43569297 | 2.29519427 |
| MYO19 | 206 | 0.00180824 | 0.44273427 | 2.25869121 |
| AURKA | 205 | 0.00173381 | 0.42026557 | 2.37944785 |
| NOTCH1 | 204 | 2.56E-04 | 0.4321124 | 2.31421268 |
| HERC2 | 204 | 0.00321709 | 0.42238922 | 2.36748466 |
| STUB1 | 202 | 4.37E-04 | 0.4229555 | 2.36431493 |
| ARRB1 | 202 | 8.92E-04 | 0.4171999 | 2.39693252 |
| FBL | 202 | 0.00201012 | 0.43917554 | 2.27699387 |
| ESR1 | 202 | 0.00137311 | 0.42146089 | 2.37269939 |
| HIST2H4B | 201 | 8.87E-04 | 0.44622895 | 2.24100204 |
| STAT1 | 200 | 0.00150288 | 0.42906028 | 2.33067485 |
| HNRNPR | 200 | 8.27E-04 | 0.40650069 | 2.46002045 |
| EPS15 | 200 | 2.27E-04 | 0.43418424 | 2.30316973 |
| NFKB1 | 200 | 0.00110018 | 0.42473725 | 2.35439673 |
| ERBB3 | 199 | 0.00105765 | 0.43104588 | 2.31993865 |
| SRPK2 | 199 | 7.43E-04 | 0.43138812 | 2.31809816 |
| SQSTM1 | 198 | 2.98E-04 | 0.43117891 | 2.3192229 |
| ARRB2 | 197 | 6.09E-04 | 0.41496945 | 2.40981595 |
| POU5F1P3 | 197 | 0.00137514 | 0.43760347 | 2.28517382 |
| SNU13 | 196 | 8.76E-04 | 0.41827046 | 2.39079755 |
| NFATC2 | 196 | 8.28E-04 | 0.41147762 | 2.43026585 |
| NF2 | 195 | 5.95E-04 | 0.43255197 | 2.31186094 |
| TRIM28 | 195 | 6.90E-04 | 0.43711451 | 2.28773006 |
| KRTAP10-3 | 195 | 0.00152106 | 0.4277841 | 2.33762781 |
| HDAC3 | 194 | 0.00123498 | 0.4171821 | 2.39703476 |
| NFKBIA | 194 | 4.03E-04 | 0.43262851 | 2.31145194 |
| ERBB2 | 194 | 0.00102068 | 0.4247926 | 2.35408998 |
| KAT2B | 194 | 0.00113661 | 0.40753396 | 2.45378323 |
| SRPK1 | 194 | 0.00196084 | 0.44229378 | 2.2609407 |
| NEDD8 | 193 | 2.50E-04 | 0.43358752 | 2.30633947 |
| AR | 193 | 0.00261067 | 0.41505751 | 2.4093047 |
| EP300 | 193 | 4.61E-04 | 0.39837067 | 2.51022495 |
| IQGAP1 | 193 | 9.90E-04 | 0.39335559 | 2.54222904 |
| SDCBP | 192 | 0.00109629 | 0.42735416 | 2.33997955 |
| NEDD4 | 192 | 8.19E-04 | 0.41375809 | 2.41687117 |
| ATXN1 | 192 | 0.00125239 | 0.42606953 | 2.34703476 |
| ACTB | 192 | 6.81E-04 | 0.44259402 | 2.25940695 |
| CCT2 | 192 | 9.02E-04 | 0.41736013 | 2.39601227 |
| HIST1H4I | 191 | 0.00104726 | 0.41769881 | 2.39406953 |
| ACTG1 | 191 | 5.19E-04 | 0.42024751 | 2.3795501 |
| PPP1CC | 190 | 7.20E-04 | 0.39780354 | 2.51380368 |
| SUZ12 | 190 | 7.15E-04 | 0.44061993 | 2.26952965 |
| PPP2CA | 190 | 4.35E-04 | 0.42966347 | 2.32740286 |
| EGFR | 190 | 7.67E-04 | 0.42536534 | 2.35092025 |
| PPP2CB | 189 | 0.00142642 | 0.44099743 | 2.26758691 |
| TUBB | 189 | 0.00183836 | 0.43569297 | 2.29519427 |
| SRRM2 | 188 | 0.00223915 | 0.45121107 | 2.21625767 |
| PPP2R1A | 188 | 7.81E-04 | 0.44456566 | 2.2493865 |
| UBL4A | 188 | 2.63E-04 | 0.43324178 | 2.30817996 |
| ACTA1 | 186 | 7.87E-04 | 0.43243721 | 2.31247444 |
| HIST3H3 | 186 | 0.00143712 | 0.43569297 | 2.29519427 |
| DDX17 | 185 | 6.85E-04 | 0.41068279 | 2.43496933 |
| RPS19 | 185 | 0.00101767 | 0.41107982 | 2.43261759 |
| RPS20 | 185 | 6.64E-04 | 0.41850315 | 2.3894683 |
| KAT5 | 185 | 1.94E-04 | 0.42140641 | 2.37300613 |
| NOP56 | 184 | 6.42E-04 | 0.4427944 | 2.25838446 |
| RPS27A | 184 | 0.00141134 | 0.40493541 | 2.46952965 |
| PPP1CA | 184 | 9.01E-04 | 0.42853387 | 2.33353783 |
| PPP1CB | 184 | 0.00118467 | 0.42207932 | 2.3692229 |
| RPS8 | 183 | 7.38E-04 | 0.41837782 | 2.39018405 |
| RPS7 | 183 | 6.90E-04 | 0.41977852 | 2.38220859 |
| RPS9 | 183 | 5.49E-04 | 0.42692509 | 2.34233129 |
| GAN | 183 | 4.21E-04 | 0.43895871 | 2.27811861 |
| SYNCRIP | 183 | 6.13E-04 | 0.42789639 | 2.33701431 |
| RPS10 | 182 | 0.0014651 | 0.40508636 | 2.46860941 |
| RPS13 | 182 | 2.12E-04 | 0.43658765 | 2.2904908 |
| RPS15A | 181 | 1.92E-04 | 0.43023051 | 2.32433538 |
| RPS11 | 181 | 0.00151711 | 0.4348406 | 2.29969325 |
| RPS14 | 181 | 4.49E-04 | 0.43754474 | 2.28548057 |
| RPS16 | 181 | 4.48E-04 | 0.43825058 | 2.28179959 |
| RPS18 | 180 | 0.00160928 | 0.42936166 | 2.32903885 |
| MMS19 | 180 | 9.10E-04 | 0.4325711 | 2.31175869 |
| RPS4X | 180 | 5.94E-04 | 0.39672238 | 2.5206544 |
| SIN3A | 180 | 5.88E-04 | 0.43571238 | 2.29509202 |
| RPS3A | 179 | 1.20E-04 | 0.4168798 | 2.39877301 |
| RPS3 | 179 | 2.23E-04 | 0.42618093 | 2.34642127 |
| RPS2 | 179 | 0.00248964 | 0.4188975 | 2.38721881 |
| RPS6 | 178 | 9.50E-04 | 0.44682018 | 2.23803681 |
| RPS5 | 178 | 4.71E-04 | 0.41395073 | 2.41574642 |
| RPS6KB2 | 178 | 4.62E-04 | 0.42438707 | 2.35633947 |
| CLTC | 178 | 0.00200935 | 0.42021139 | 2.3797546 |
| CUL5 | 178 | 8.34E-04 | 0.43689971 | 2.28885481 |
| POT1 | 178 | 5.78E-04 | 0.40159323 | 2.4900818 |
| WWOX | 177 | 1.66E-04 | 0.4224622 | 2.36707566 |
| RACK1 | 177 | 9.34E-04 | 0.43672412 | 2.28977505 |
| PRMT5 | 177 | 9.96E-04 | 0.428985 | 2.33108384 |
| COMMD3-BMI1 | 177 | 7.57E-04 | 0.43445427 | 2.30173824 |
| RPL14 | 177 | 5.57E-04 | 0.43884053 | 2.27873211 |
| SHC1 | 176 | 2.34E-04 | 0.4292486 | 2.32965235 |
| C1QBP | 176 | 0.00127906 | 0.39104358 | 2.55725971 |
| HDAC5 | 176 | 9.43E-04 | 0.4130242 | 2.42116564 |
| OFD1 | 176 | 7.49E-04 | 0.43157848 | 2.31707566 |
| TRIM27 | 175 | 6.67E-04 | 0.41578097 | 2.40511247 |
| BRCA1 | 175 | 6.82E-04 | 0.43941232 | 2.27576687 |
| SIRT7 | 175 | 0.00235878 | 0.4282524 | 2.33507157 |
| TARDBP | 175 | 6.25E-04 | 0.42224333 | 2.36830266 |
| SF3B1 | 174 | 0.00171504 | 0.42752229 | 2.3390593 |
| KRT40 | 174 | 0.00138101 | 0.42512497 | 2.35224949 |
| REL | 174 | 6.47E-04 | 0.41807378 | 2.39192229 |
| SF3B3 | 173 | 0.00123632 | 0.4128847 | 2.42198364 |
| RELA | 173 | 0.00140027 | 0.41911292 | 2.38599182 |
| MAP3K3 | 172 | 7.98E-04 | 0.44245385 | 2.2601227 |
| MAP3K1 | 172 | 3.55E-04 | 0.42013919 | 2.3801636 |
| CBX5 | 172 | 0.00103363 | 0.42979565 | 2.32668712 |
| HDAC6 | 172 | 8.26E-04 | 0.42084427 | 2.37617587 |
| DDX5 | 171 | 3.25E-04 | 0.42078995 | 2.37648262 |
| RB1 | 171 | 3.73E-04 | 0.4363539 | 2.29171779 |
| DDX3X | 171 | 0.00121345 | 0.41805591 | 2.39202454 |
| RBBP4 | 171 | 0.00166757 | 0.42958798 | 2.32781186 |
| DHX9 | 170 | 2.94E-04 | 0.39307102 | 2.54406953 |
| RBBP7 | 170 | 8.62E-04 | 0.41484624 | 2.4105317 |
| DHX15 | 170 | 6.86E-04 | 0.42577275 | 2.34867076 |
| CUL4A | 170 | 0.00105593 | 0.45127353 | 2.21595092 |
| RBX1 | 170 | 7.31E-04 | 0.40129662 | 2.49192229 |
| SIRT1 | 169 | 9.51E-04 | 0.43218878 | 2.31380368 |
| CUL4B | 169 | 5.75E-04 | 0.42089861 | 2.37586912 |
| CUL1 | 169 | 7.87E-04 | 0.4348406 | 2.29969325 |
| CUL2 | 169 | 3.17E-04 | 0.41698644 | 2.39815951 |
| MDM2 | 169 | 1.58E-04 | 0.42330332 | 2.36237219 |
| BMI1 | 169 | 0.00364499 | 0.4193825 | 2.38445808 |
| CUL3 | 168 | 4.94E-04 | 0.39049711 | 2.56083845 |
| PAN2 | 168 | 5.67E-04 | 0.41489903 | 2.41022495 |
| DDB1 | 168 | 0.0010785 | 0.43364519 | 2.30603272 |
| DCTN1 | 168 | 1.45E-04 | 0.41807378 | 2.39192229 |
| LIMA1 | 168 | 3.46E-04 | 0.42759706 | 2.33865031 |
| LGALS3 | 167 | 6.71E-04 | 0.43760347 | 2.28517382 |
| TMEM17 | 167 | 8.08E-04 | 0.43553774 | 2.29601227 |
| ZDHHC17 | 167 | 0.00111162 | 0.42977676 | 2.32678937 |
| HIST1H4B | 167 | 2.56E-04 | 0.42866535 | 2.33282209 |
| RAD21 | 167 | 1.75E-04 | 0.43030623 | 2.32392638 |
| HIST1H4C | 166 | 2.96E-04 | 0.42853387 | 2.33353783 |
| HIST1H4E | 166 | 3.94E-04 | 0.43768181 | 2.28476483 |
| HIST2H4A | 166 | 5.51E-04 | 0.4172889 | 2.39642127 |
| HIST1H4H | 165 | 7.67E-04 | 0.44561899 | 2.24406953 |
| HIST1H4L | 165 | 7.01E-04 | 0.42372514 | 2.36002045 |
| DAXX | 165 | 1.10E-04 | 0.42253521 | 2.36666667 |
| OBSL1 | 165 | 0.00127574 | 0.3897812 | 2.56554192 |
| RAF1 | 164 | 0.0020682 | 0.43429992 | 2.30255624 |
| DBN1 | 164 | 4.10E-04 | 0.43091294 | 2.3206544 |
| XRCC5 | 164 | 6.16E-04 | 0.43656816 | 2.29059305 |
| HNRNPM | 164 | 2.21E-04 | 0.43251371 | 2.31206544 |
| XPO1 | 163 | 9.95E-04 | 0.42685056 | 2.34274029 |
| YWHAE | 163 | 8.32E-04 | 0.40736421 | 2.45480573 |
| YWHAG | 163 | 1.83E-04 | 0.43497598 | 2.29897751 |
| NEDD4L | 163 | 0.00167526 | 0.42701829 | 2.34182004 |
| YWHAB | 162 | 5.12E-04 | 0.42972011 | 2.32709611 |
| YWHAH | 162 | 6.66E-04 | 0.40987385 | 2.43977505 |
| NCK1 | 162 | 9.80E-04 | 0.3995098 | 2.50306748 |
| SF1 | 162 | 3.42E-04 | 0.39788446 | 2.51329243 |
| RAC1 | 162 | 6.52E-04 | 0.39785209 | 2.51349693 |
| APP | 162 | 6.06E-04 | 0.42217042 | 2.36871166 |
| YWHAZ | 161 | 5.12E-04 | 0.42936166 | 2.32903885 |
| NCL | 161 | 4.44E-04 | 0.43280081 | 2.3105317 |
| HNRNPK | 161 | 6.26E-04 | 0.42095295 | 2.37556237 |
| MYO1C | 161 | 0.00166093 | 0.43809353 | 2.28261759 |
| FBXW11 | 161 | 3.77E-04 | 0.44107699 | 2.26717791 |
| PXN | 161 | 3.69E-04 | 0.40223739 | 2.48609407 |
| APC | 161 | 8.74E-04 | 0.42251696 | 2.36676892 |
| DCUN1D1 | 160 | 1.86E-04 | 0.42466348 | 2.35480573 |
| CUL7 | 160 | 2.66E-04 | 0.41760963 | 2.39458078 |
| MYH9 | 160 | 1.83E-04 | 0.42361502 | 2.36063395 |
| HNRNPC | 159 | 9.34E-04 | 0.43437708 | 2.30214724 |
| HNRNPA1 | 159 | 4.62E-04 | 0.43600374 | 2.29355828 |
| HNRNPH1 | 159 | 7.63E-04 | 0.44285456 | 2.25807771 |
| HNRNPA2B1 | 158 | 3.38E-04 | 0.39310262 | 2.54386503 |
| HNRNPD | 158 | 9.28E-05 | 0.42302868 | 2.36390593 |
| EEF1A1 | 158 | 9.68E-04 | 0.40612931 | 2.46226994 |
| VCP | 158 | 6.89E-04 | 0.40449996 | 2.47218814 |
| MYC | 158 | 7.78E-04 | 0.37814639 | 2.64447853 |
| PTPN11 | 158 | 8.32E-04 | 0.40816327 | 2.45 |
| EEF1G | 158 | 0.00121886 | 0.41458245 | 2.41206544 |
| EEF2 | 158 | 4.48E-04 | 0.44119637 | 2.26656442 |
| HDAC4 | 158 | 0.00114668 | 0.43617875 | 2.29263804 |
| VHL | 157 | 3.91E-04 | 0.39527928 | 2.52985685 |
| VIM | 157 | 7.47E-04 | 0.44032236 | 2.27106339 |
| EZR | 157 | 7.32E-04 | 0.43919526 | 2.27689162 |
| HIST4H4 | 157 | 8.51E-04 | 0.41447703 | 2.41267894 |
| VCAM1 | 157 | 3.80E-04 | 0.45064971 | 2.2190184 |
| VCL | 156 | 7.23E-05 | 0.42138825 | 2.37310838 |
| HIF1A | 156 | 5.57E-04 | 0.4137931 | 2.41666667 |
| HUWE1 | 156 | 3.85E-04 | 0.38826472 | 2.57556237 |
| HLA-B | 156 | 7.05E-04 | 0.42812117 | 2.33578732 |
